# Supplementary material for: Novel Set of Diarylmethanes to Target Colorectal Cancer: Synthesis, In Vitro and In Silico Studies
Source: Biomolecules. 2022 Dec 27;13(1):54. doi: 10.3390/biom13010054 (PMC9855432; doi:10.3390/biom13010054)
Supplement: Supplementary file 1 [file biomolecules-13-00054-s001.zip › biomolecules-2109586-SI-1.pdf]

Novel set of diarylmethanes to target colorectal cancer: synthesis, *in vitro* and *in silico* studies

Ameni Hadj Mohamed [a] [b], Aline Pinon [c], Nathalie Lagarde [a], Elizabeth Goya Jorge[d], Hadley Mouhsine[e], Moncef Msaddek[b], Bertrand Liagre *†[c], Maité Sylla-Iyarreta Veitía *† [a]

a. Laboratoire Génomique, Bioinformatique et Chimie Moléculaire (GBCM, EA 7528) Conservatoire national des arts et métiers, HESAM Université, 2 rue Conté, 75003, Paris
b. Laboratoire de Chimie hétérocyclique, produits naturels et réactivité (LR11ES39) Université de Monastir Avenue de l'environnement 5019 Monastir, Tunisie
Univ. Limoges, LABCiS, UR 22722, F-87000 Limoges, France
c. Département de sciences des denrées alimentaires, Faculté de Médecine vétérinaire (DDA) – FARAH, Université de Liège, avenue de Cureghem 10 4000 Liège 1, Belgique
d. Peptinov, Pépinière Paris Santé Cochin, Hôpital Cochin, 29 rue du Faubourg Saint Jacques Paris 75014

*Corresponding authors: Pr. M. Sylla-Iyarreta Veitía, maite.sylla@lecnam.net;
Pr. B. Liagre, bertrand.liagre@unilim.fr

† These authors contributed equally to the work.

Synthesis and characterization of intermediates

(4-methoxyphenyl)(pyridin-2-yl) methanol 3

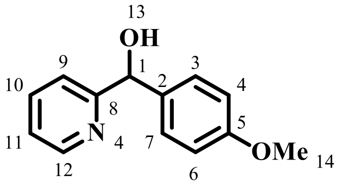

<u>Method 1</u>

To a solution of 2-bromopyridine (25 mmol, 2.38 mL) in anhydrous THF (25 mL) at -78 °C, was added dropwise *n*-BuLi (1M) solution in hexane (30 mmol, 30 mL, 1.2 eq) under argon. The mixture was stirred during 1 h 30 min at -78 °C and *p*-anisaldehyde (27.5 mmol, 3.45 mL, 1.1 eq) was then added dropwise and the reaction was stirred at room temperature during 6 days. After reaction completion, the mixture was poured into the water and extracted with DCM. The combined organic extracts were dried over anhydrous $MgSO_4$, filtered and concentrated under vacuum. The oily crude was purified by FCC on silica gel (CyHex/ EtOAc, 50:50) to afford the (4-methoxyphenyl)(pyridin-2-yl)methanol **3** as a white solid in 22% yield (1163 mg). A by-product **4** was also isolated as a white solid in 2% yield (96 mg).

<u>Method 2</u>

To a solution of 2-bromopyridine (10 mmol, 0.97 mL) in anhydrous THF (10 mL), was added 2M *iPr*MgCl solution in THF (1 eq, 10 mmol, 5 mL) under argon. The reaction was stirred at room temperature during 2 h and *p*-anisaldehyde (1.2 eq, 12 mmol, 1.48 mL) was added then dropwise and the reaction was stirred at room temperature during 2 h.

After reaction completion, the mixture was poured into the water and extracted with DCM. The combined organic extracts were dried over anhydrous $MgSO_4$, filtered and concentrated under vacuum. The oily crude was purified by FCC on silica gel (CyHex/EtOAc, 50:50) to afford the desired compound **3** as a white solid in 63% yield (1.33 g).

TLC: Cyhex/EtOAc: 50:50, R_f = 0.3

^{1}H NMR (400 MHz, DMSO-*d*$_6$) *δ* (ppm) : 8.43 (ddd, $J_{12\text{-}11}$ = 4.7 Hz, $J_{12\text{-}10}$ = 1.9 Hz, $J_{12\text{-}9}$ = 0.9 Hz, 1H, H12), 7.76 (td, $J_{10\text{-}11,\ 10\text{-}9}$ = 7.7 Hz, $J_{10\text{-}12}$ = 1.8 Hz, 1H, H10), 7.53 (d, $J_{9\text{-}10}$ = 7.9 Hz, 1H, H9), 7.29 – 7.26 (m, 2H, H3, H7), 7.22 - 7.19 (m, 1H, H11), 6.86-6.82 (m, 2H, H4, H6), 5.95 (d, $J_{1\text{-}13}$ = 4.2 Hz, 1H, H1), 5.65 (d, $J_{13\text{-}1}$ = 4.2 Hz, 1H, OH), 3.69 (s, 3H, CH_3).

^{13}C NMR (100 MHz, DMSO-*d6*) *δ* (ppm) : 164.7 (C5), 158.69 (C8), 148.7 (C12), 137.2 (C10), 136.9 (C2), 128.0 (C3, C7), 122.4 (C9), 120.3 (C11), 113.8 (C4, C6), 75.6 (C13), 55.7 (C1).

LRMS: (ES+, CV=30) m/z: 215.23 $[M]^{+}$; 214.15 $[M\text{-}H]^{+}$; 198.16 $[M\text{-}OH]^{+}$.

IR ν (cm^{-1}) : 3170 (ν_{OH}); 3078, 3015 ($\nu_{Csp2\text{-}H}$); 2962 ($\nu_{Csp3\text{-}H)}$; 2840 ($\nu_{O\text{-}CH3}$); 1593, 1591, 1514 and 1465 ($\nu_{C=C}$); 1256 ($\nu_{C\text{-}O}$); 817 ($\delta_{Csp2\text{-}H\ p\text{-}substitution}$).

1,2-bis(4-methoxyphenyl)-1-(pyridin-2-yl)ethane-1,2-diol 4

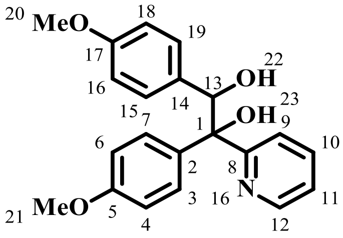

$C_{21}H_{21}NO_4$
351.40 g/mol

TLC: Cyhex/EtOAc: 50:50, R_f = 0.5

^{1}H NMR (400 MHz, DMSO-d_6) δ (ppm) : 8.57 (ddd, $J_{12\text{-}11}$ = 4.7 Hz, $J_{12\text{-}10}$ = 1.9 Hz, $J_{12\text{-}9}$ = 0.9 Hz, 1H, H12), 7.76 – 7.69 (m, 2H, 2H, H9, H10), 7.36 - 7.34 (m, 2H, H14, H18), 7.25 – 7.23 (m, 1H, H11), 7.09 (d, $J_{3\text{-}4,\ 7\text{-}8}$ = 8.6 Hz, 2H, H3, H7), 6.68-6.62 (m, 4H, H4, H6, H15, H17), 5.72 (s, 1H, H23, OH), 5.69 (d, $J_{13\text{-}22}$ = 5.3 Hz, 1H, H13), 5.41 (d, $J_{22\text{-}13}$ = 5.3 Hz,1H, OH) 3.64 (s, 3H, CH_3), 3.62 (s, 3H, CH_3).

^{13}C NMR (100 MHz, DMSO-d_6) δ (ppm) : 165.8 (C8), 158.2 (C5), 157.9 (C17), 147.7 (C12), 137.01 (C10), 136.7 (C14), 134.3 (C2), 130.15 (C15, C19), 127.76 (C3, C7), 122.03 (C9), 121.77 (C11), 113.04 (C4, C6), 112.54 (C16, C18), 80.64 (C1), 77.3 (C13), 55.27 (C21), 55.25 (C20).

LRMS: (ES+, CV=30). m/z : 352 $[M+H]^{+}$, 374 $[M+23+H]^{+}$; 334 $[M\text{-}OH]^{+}$.

IR ν (cm^{-1}) : 3375 (ν_{OH}), 3113, 3005 ($\nu_{Csp2\text{-}H}$); 2972, 2932 ($\nu_{Csp3\text{-}H)}$; 2840 (ν_{OMe}); 1610, 1511 and 1461 ($\nu_{C=C}$); 1173 ($\nu_{C\text{-}O)}$; 840 ($\delta_{Csp2\text{-}H\ p\text{-}substitution}$).

HRMS: calcd. for $C_{21}H_{21}NO_4H$ $[M+H]^{+}$ (352.1543); found (353.1385).
