# Supplementary material for: Novel Set of Diarylmethanes to Target Colorectal Cancer: Synthesis, In Vitro and In Silico Studies
Source: Biomolecules. 2022 Dec 27;13(1):54. doi: 10.3390/biom13010054 (PMC9855432; doi:10.3390/biom13010054)
Supplement: Supplementary file 1 [file biomolecules-13-00054-s001.zip › biomolecules-2109586-SI-2.pdf]

SI-2: Cytotoxicity evaluation

Novel set of diarylmethanes to target colorectal cancer: synthesis, *in vitro* and *in silico* studies

Ameni Hadj Mohamed [a] [b], Aline Pinon [c], Nathalie Lagarde [a], Elizabeth Goya Jorge[d], Hadley Mouhsine[e], Moncef Msaddek[b], Bertrand Liagre *†[c], Maité Sylla-Iyarreta Veitía *† [a]

a. Laboratoire Génomique, Bioinformatique et Chimie Moléculaire (GBCM, EA 7528) Conservatoire national des arts et métiers, HESAM Université, 2 rue Conté, 75003, Paris
b. Laboratoire de Chimie hétérocyclique, produits naturels et réactivité (LR11ES39) Université de Monastir Avenue de l'environnement 5019 Monastir, Tunisie
   Univ. Limoges, LABCiS, UR 22722, F-87000 Limoges, France
c. Département de sciences des denrées alimentaires, Faculté de Médecine vétérinaire (DDA) – FARAH, Université de Liège, avenue de Cureghem 10 4000 Liège 1, Belgique
d. Peptinov, Pépinière Paris Santé Cochin, Hôpital Cochin, 29 rue du Faubourg Saint Jacques Paris 75014

*Corresponding authors: Pr. M. Sylla-Iyarreta Veitía, maite.sylla@lecnam.net;

Pr. B. Liagre, bertrand.liagre@unilim.fr

† These authors contributed equally to the work.

Cytotoxicity evaluation

80% confluent L929 cells were plated in flat bottom plates at 4 x 10^5 cells per well in 100 µL of DMEM medium containing 10% FBS, 2 mM L-Glutamine, 100 U/mL Penicillin - 100 µg/mL Streptomycin (complete medium) and incubated for one night at 37 °C, 5% CO_2. Serial dilutions of compounds (from 100 µM to 0.78 µM) diluted in complete medium containing 0.5 % DMSO were added to the wells and incubated for 24 hours. Supernatants were discarded and 100 µL of MTT at 0.5 mg/mL were added to the wells. After 2 hours, supernatants were discarded and 200 µL of DMSO were added. Plates were read at 570 nm with a spectrophotometer providing the optical density (OD) of each well and percentages of survival were calculated for each well using the formula:

$$Percentage\ of\ survival = \frac{ODcompound}{OD\ negative\ control} \times 100$$

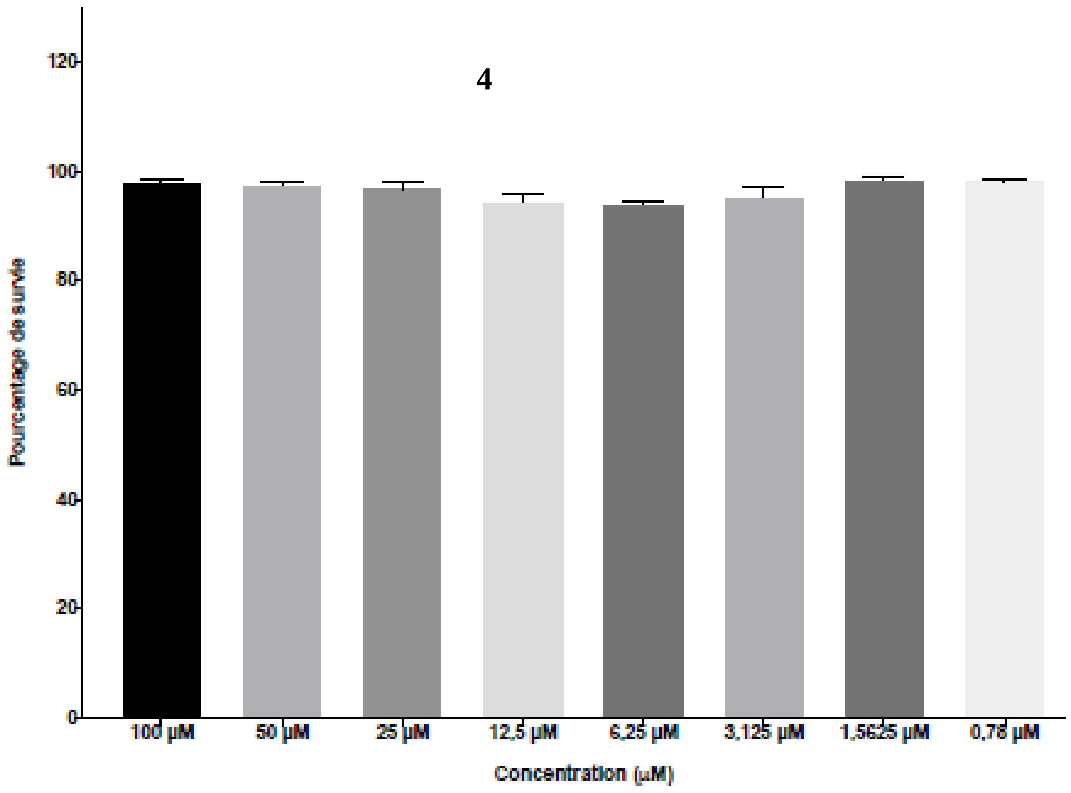

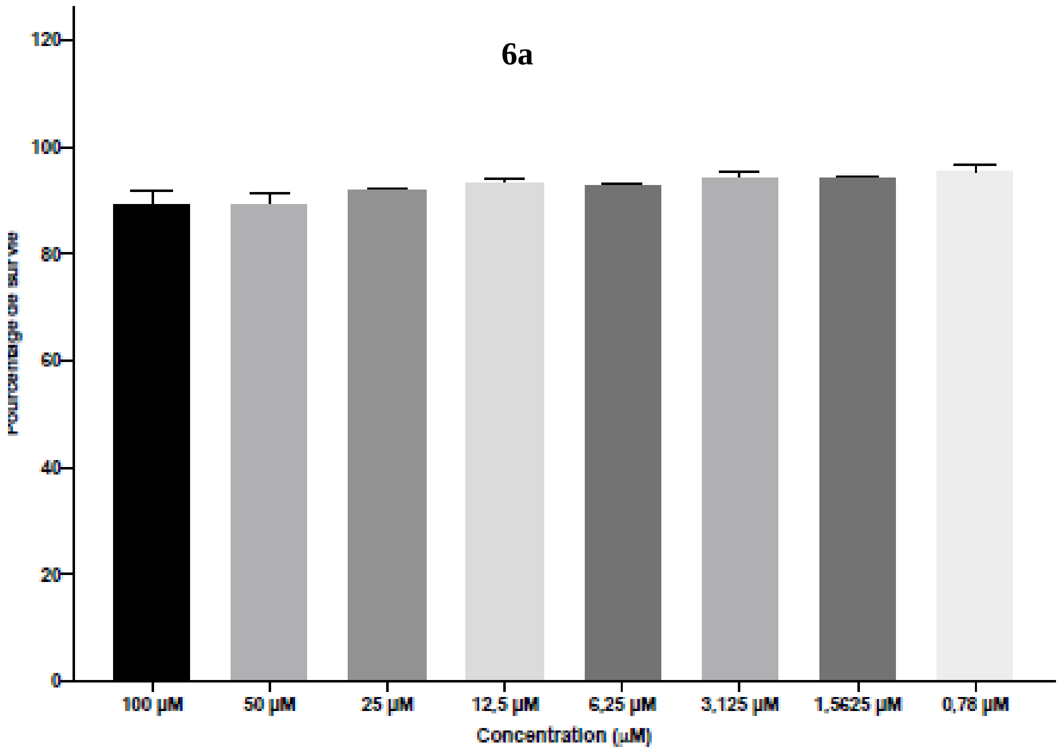

6a
Pourcentage de survie
120
100
80
60
40
20
0
100 µM
50 µM
25 µM
12,5 µM
6,25 µM
3,125 µM
1,5625 µM
0,78 µM
Concentration (µM)

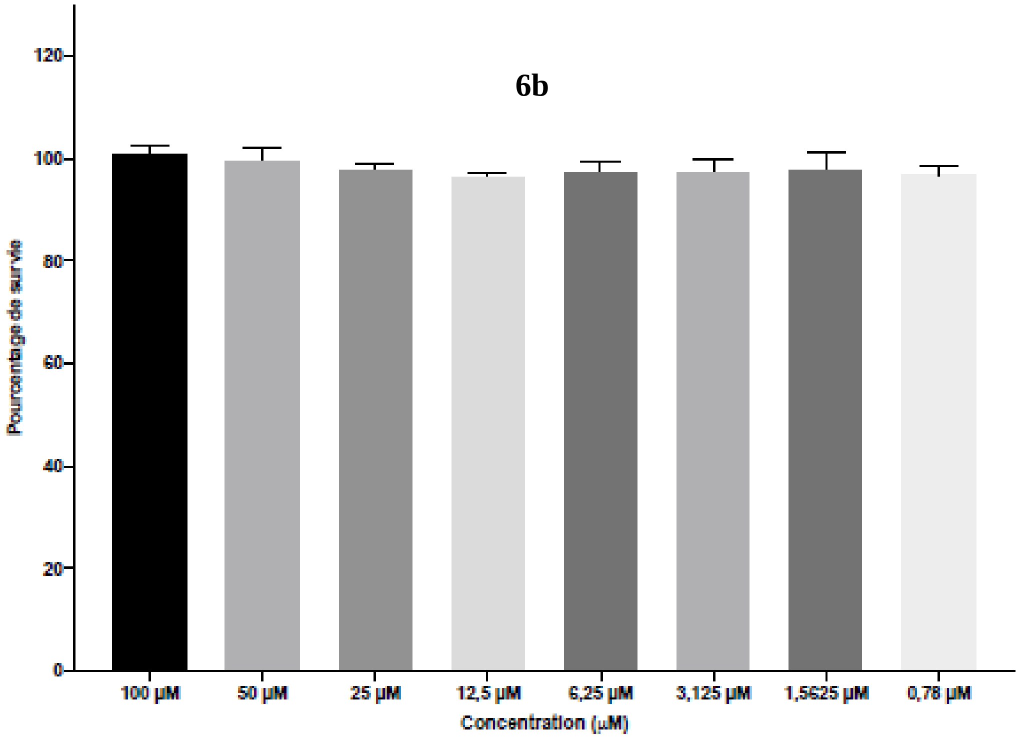

6b
Pourcentage de survie
120
100
80
60
40
20
0
100 µM
50 µM
25 µM
12,5 µM
6,25 µM
3,125 µM
1,5625 µM
0,78 µM
Concentration (µM)

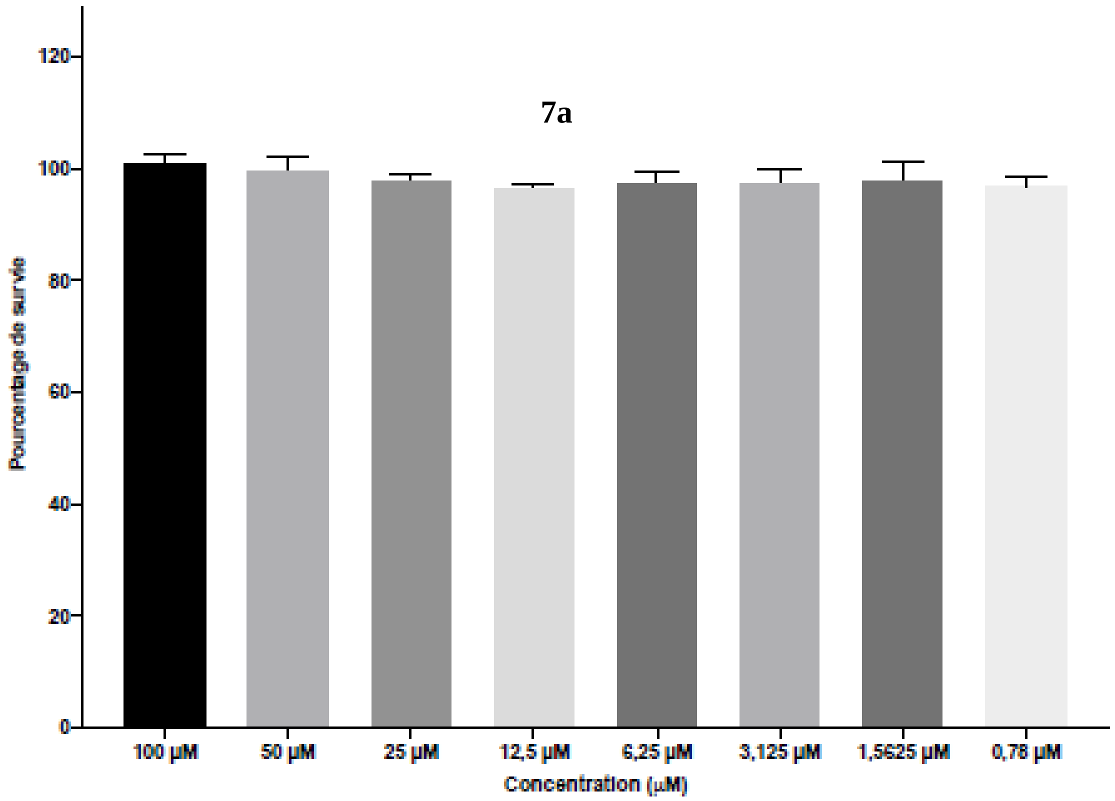
7a
120
100
80
60
40
20
0
Pourcentage de survie
100 µM
50 µM
25 µM
12,5 µM
6,25 µM
3,125 µM
1,5625 µM
0,78 µM
Concentration (µM)

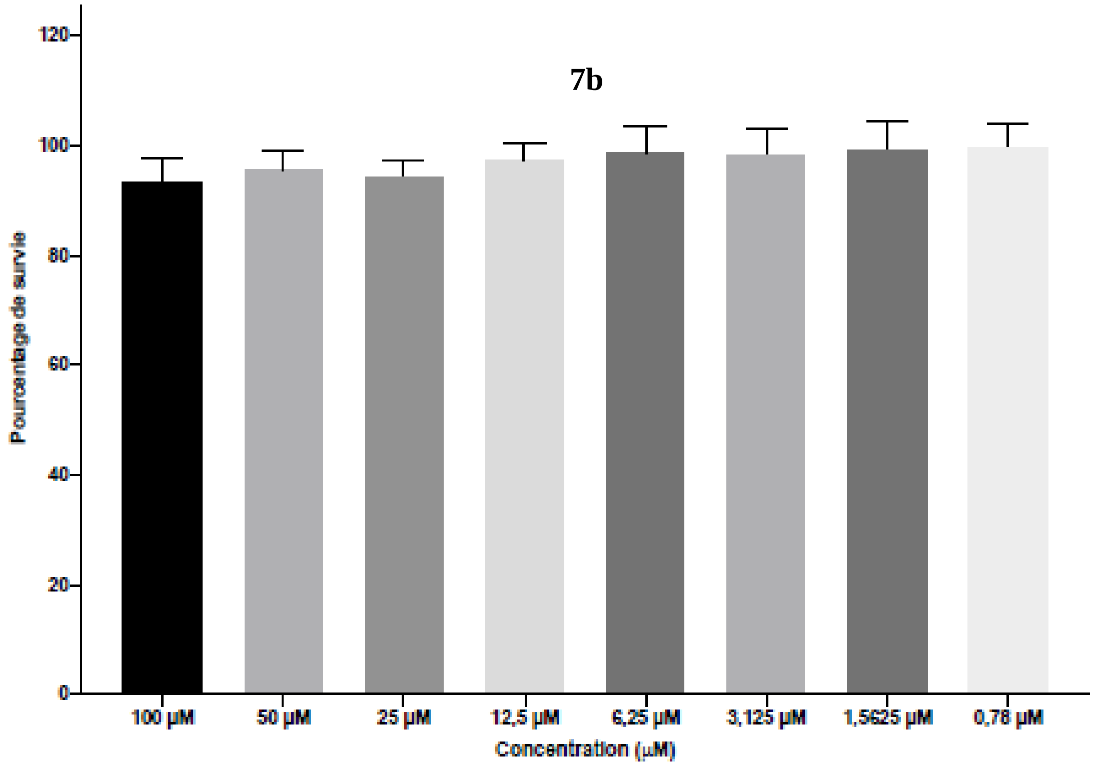
7b
120
100
80
60
40
20
0
Pourcentage de survie
100 µM
50 µM
25 µM
12,5 µM
6,25 µM
3,125 µM
1,5625 µM
0,78 µM
Concentration (µM)

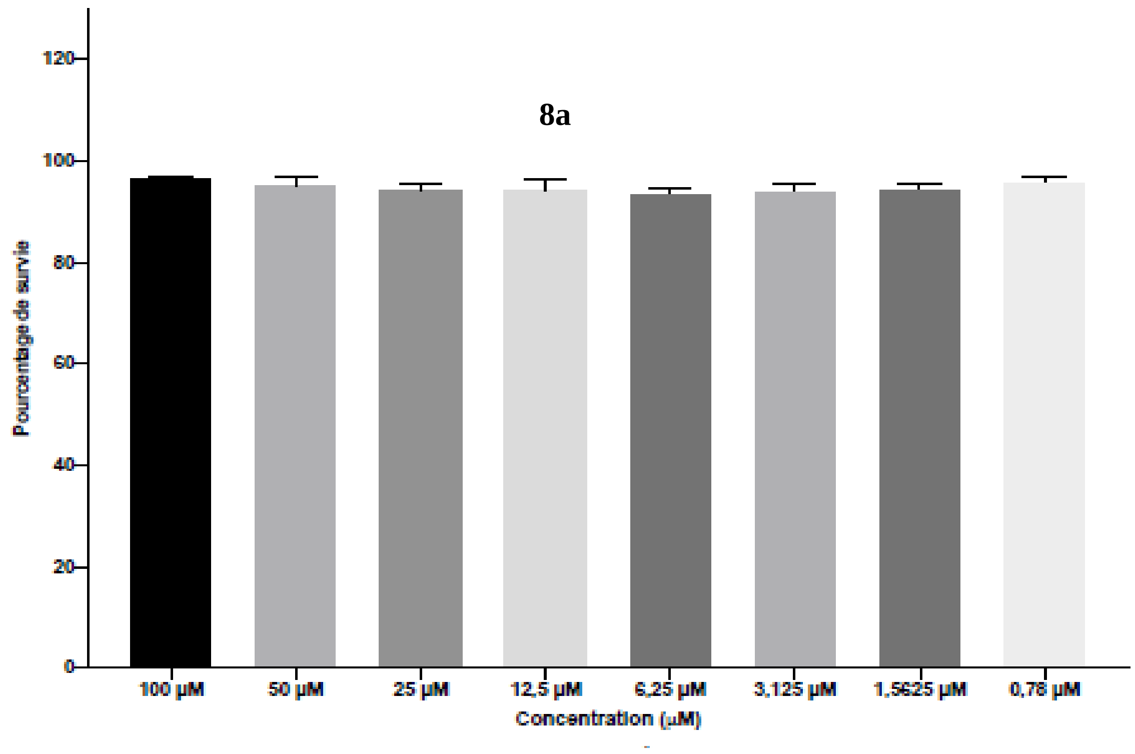
8a
Pourcentage de survie
120
100
80
60
40
20
0
100 µM
50 µM
25 µM
12,5 µM
6,25 µM
3,125 µM
1,5625 µM
0,78 µM
Concentration (µM)

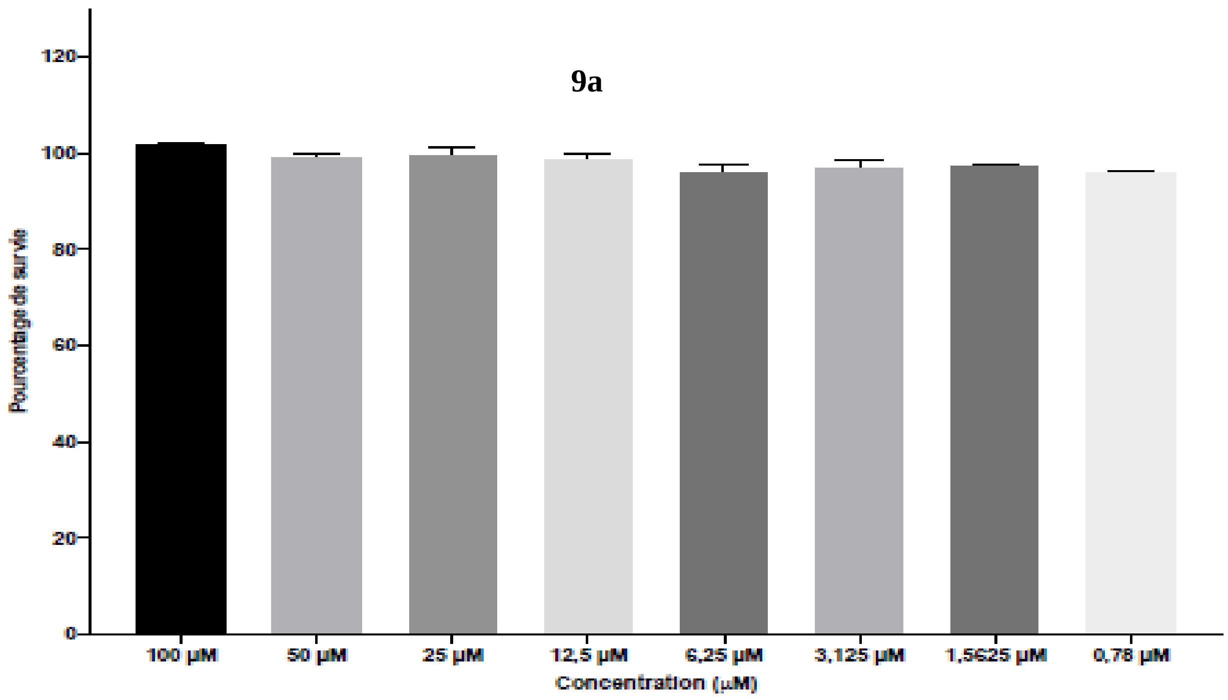
9a
Pourcentage de survie
120
100
80
60
40
20
0
100 µM
50 µM
25 µM
12,5 µM
6,25 µM
3,125 µM
1,5625 µM
0,78 µM
Concentration (µM)

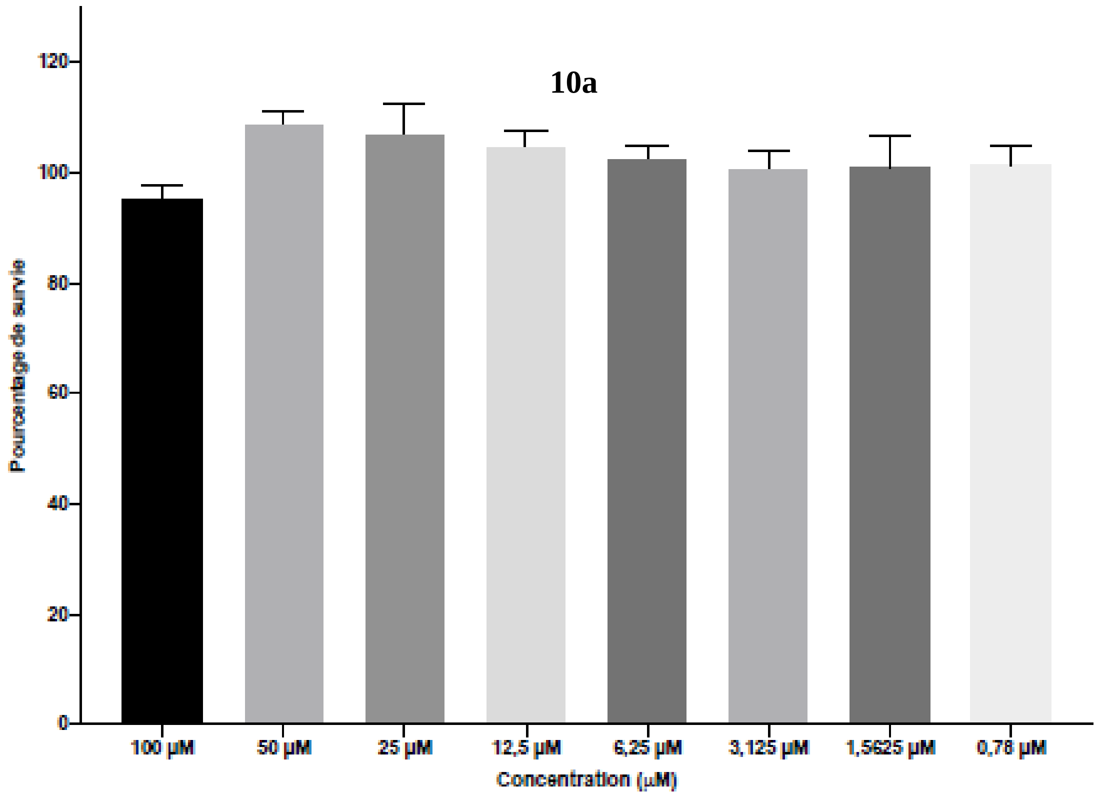
10a
Pourcentage de survie
120
100
80
60
40
20
0
100 µM
50 µM
25 µM
12,5 µM
6,25 µM
3,125 µM
1,5625 µM
0,78 µM
Concentration (µM)

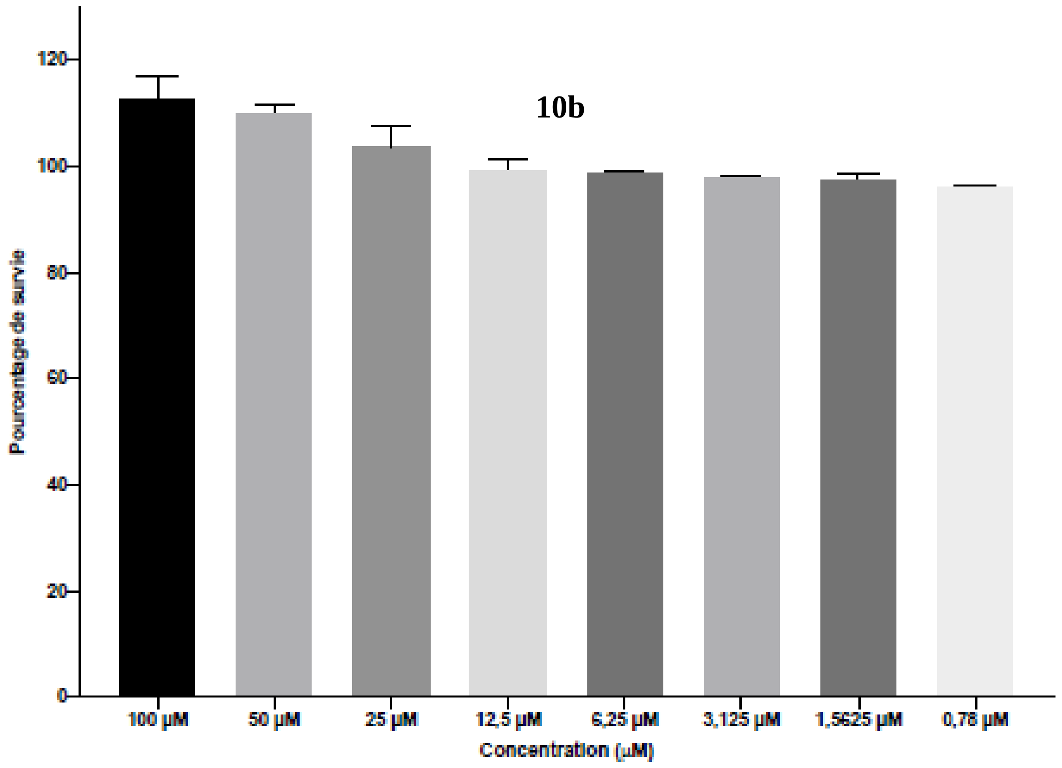
10b
Pourcentage de survie
120
100
80
60
40
20
0
100 µM
50 µM
25 µM
12,5 µM
6,25 µM
3,125 µM
1,5625 µM
0,78 µM
Concentration (µM)

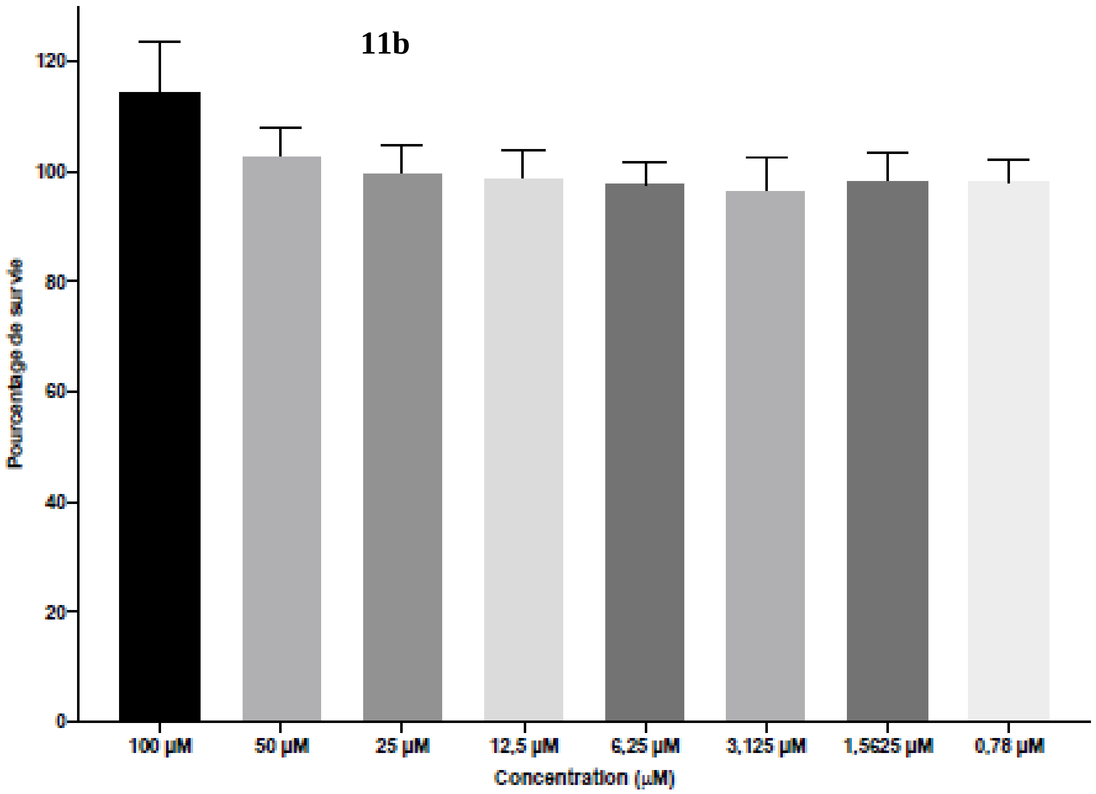
11b
Pourcentage de survie
120
100
80
60
40
20
0
100 µM
50 µM
25 µM
12,5 µM
6,25 µM
3,125 µM
1,5625 µM
0,78 µM
Concentration (µM)

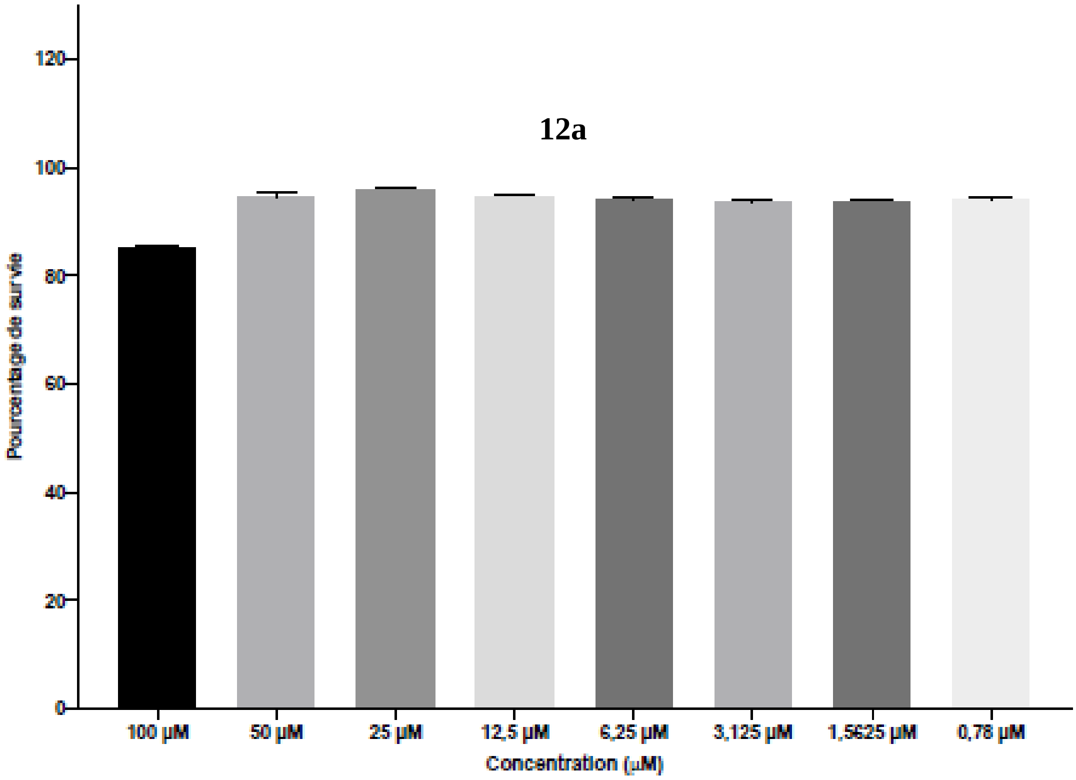
12a
Pourcentage de survie
120
100
80
60
40
20
0
100 µM
50 µM
25 µM
12,5 µM
6,25 µM
3,125 µM
1,5625 µM
0,78 µM
Concentration (µM)

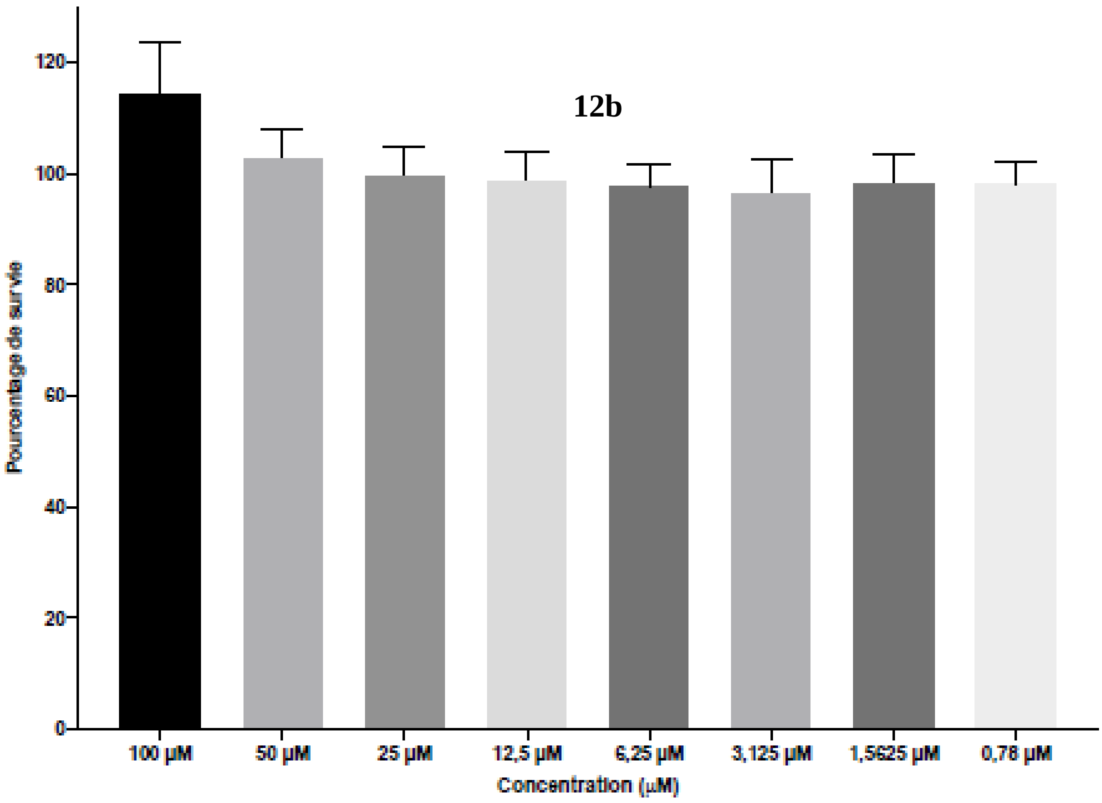
12b
Pourcentage de survie
120
100
80
60
40
20
0
100 µM
50 µM
25 µM
12,5 µM
6,25 µM
3,125 µM
1,5625 µM
0,78 µM
Concentration (µM)

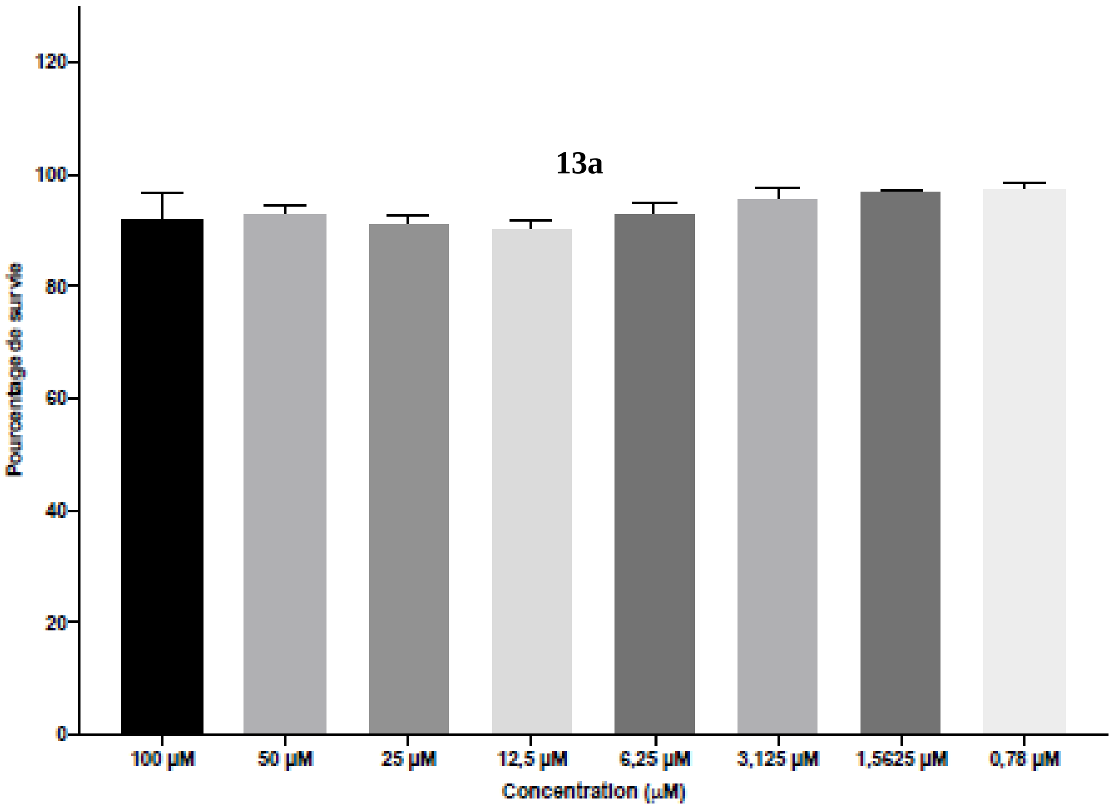
13a
Pourcentage de survie
120
100
80
60
40
20
0
100 µM
50 µM
25 µM
12,5 µM
6,25 µM
3,125 µM
1,5625 µM
0,78 µM
Concentration (µM)

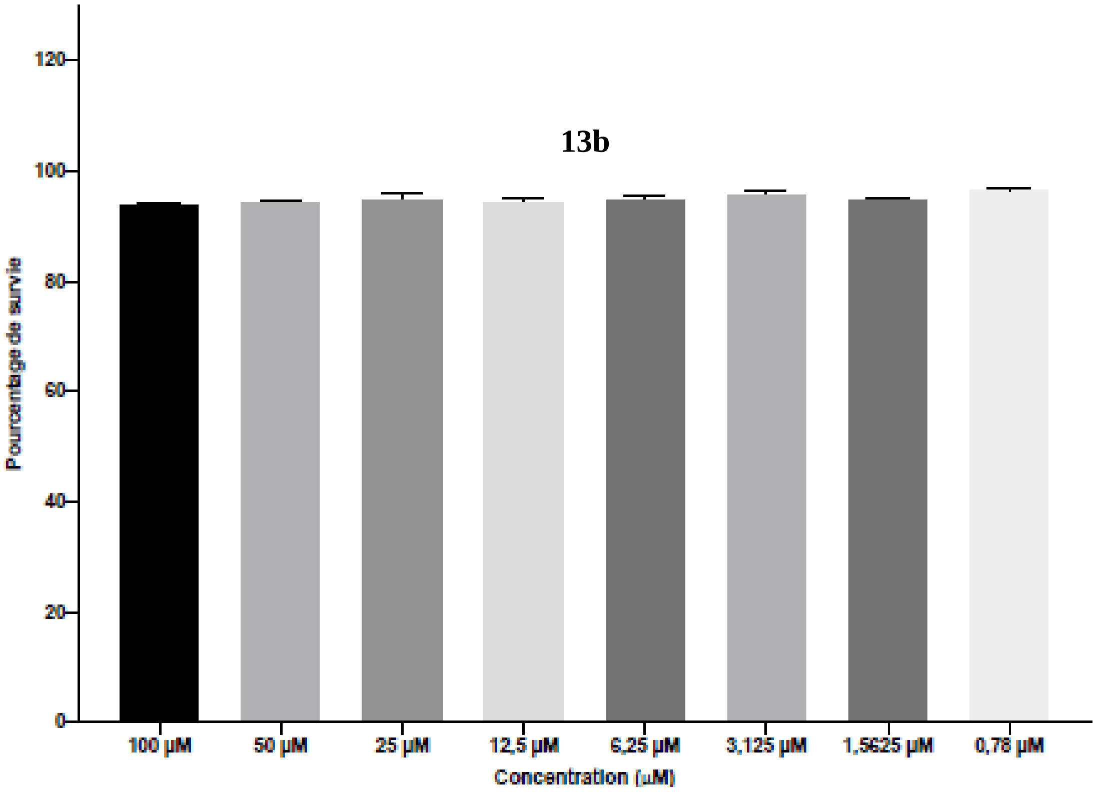
13b
Pourcentage de survie
120
100
80
60
40
20
0
100 µM
50 µM
25 µM
12,5 µM
6,25 µM
3,125 µM
1,5625 µM
0,78 µM
Concentration (µM)

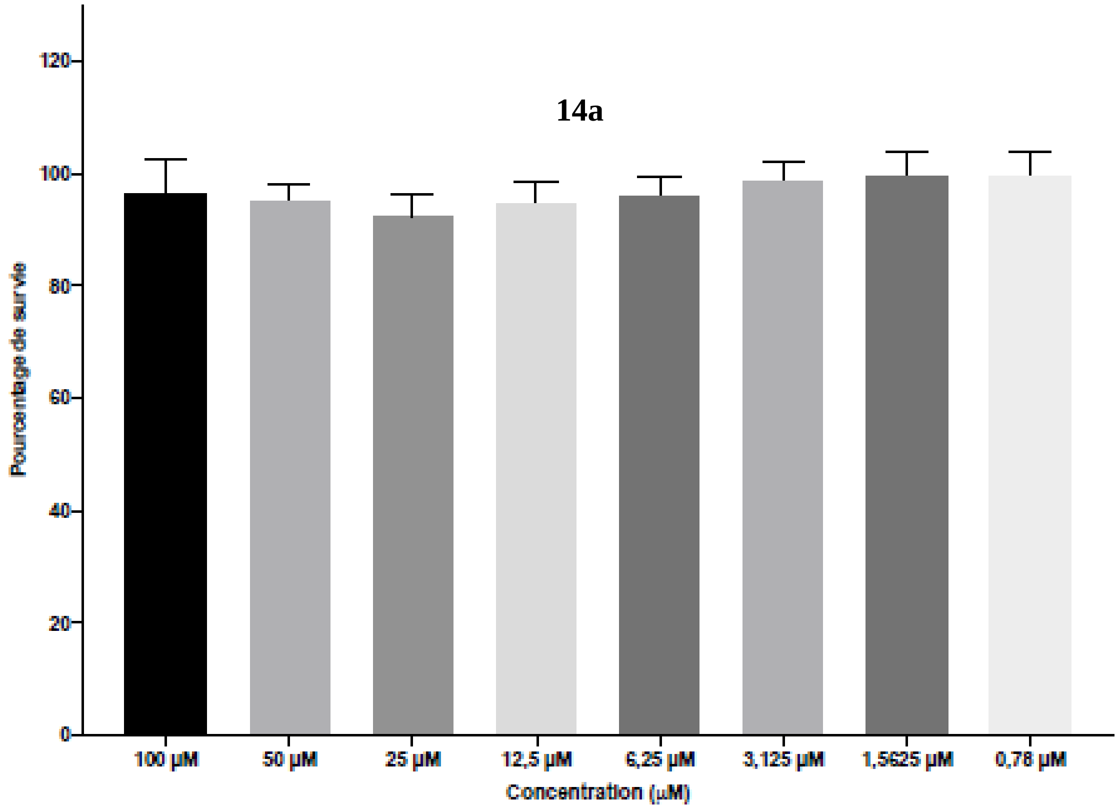
14a
120
100
80
60
40
20
0
Pourcentage de survie
100 µM
50 µM
25 µM
12,5 µM
6,25 µM
3,125 µM
1,5625 µM
0,78 µM
Concentration (µM)

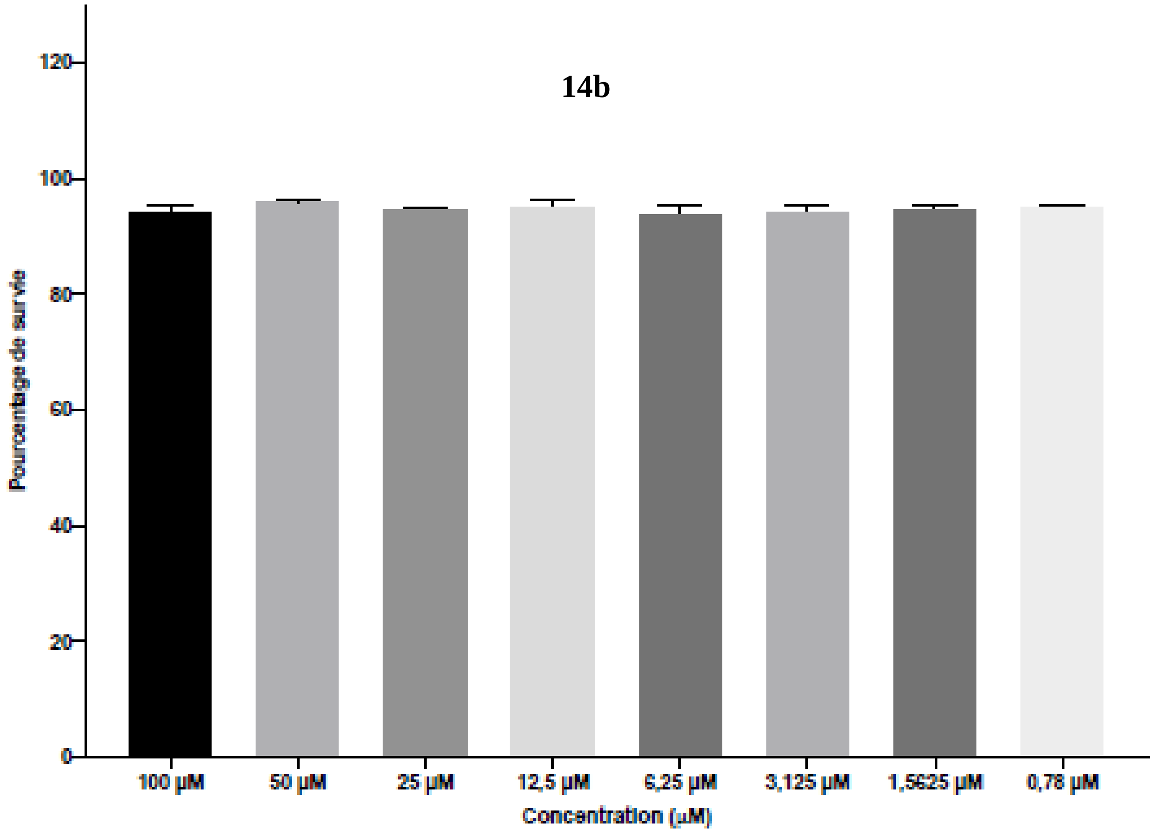
14b
120
100
80
60
40
20
0
Pourcentage de survie
100 µM
50 µM
25 µM
12,5 µM
6,25 µM
3,125 µM
1,5625 µM
0,78 µM
Concentration (µM)

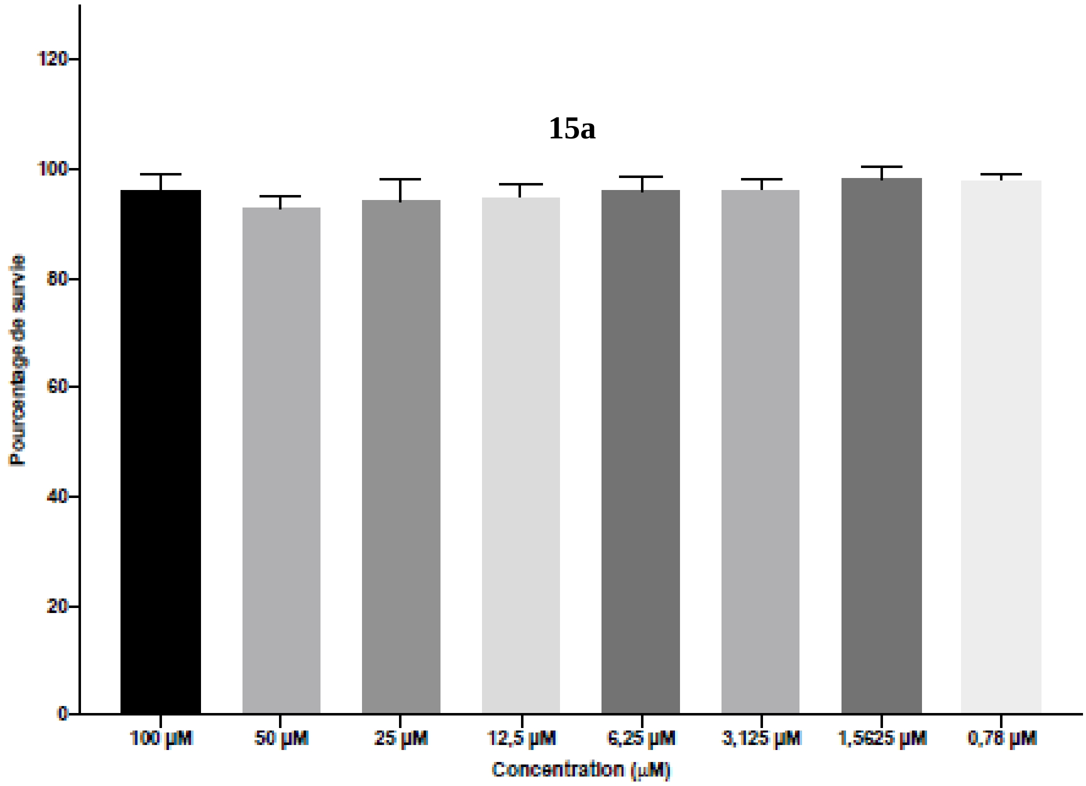
15a
Pourcentage de survie
0
20
40
60
80
100
120
100 µM
50 µM
25 µM
12,5 µM
6,25 µM
3,125 µM
1,5625 µM
0,78 µM
Concentration (µM)

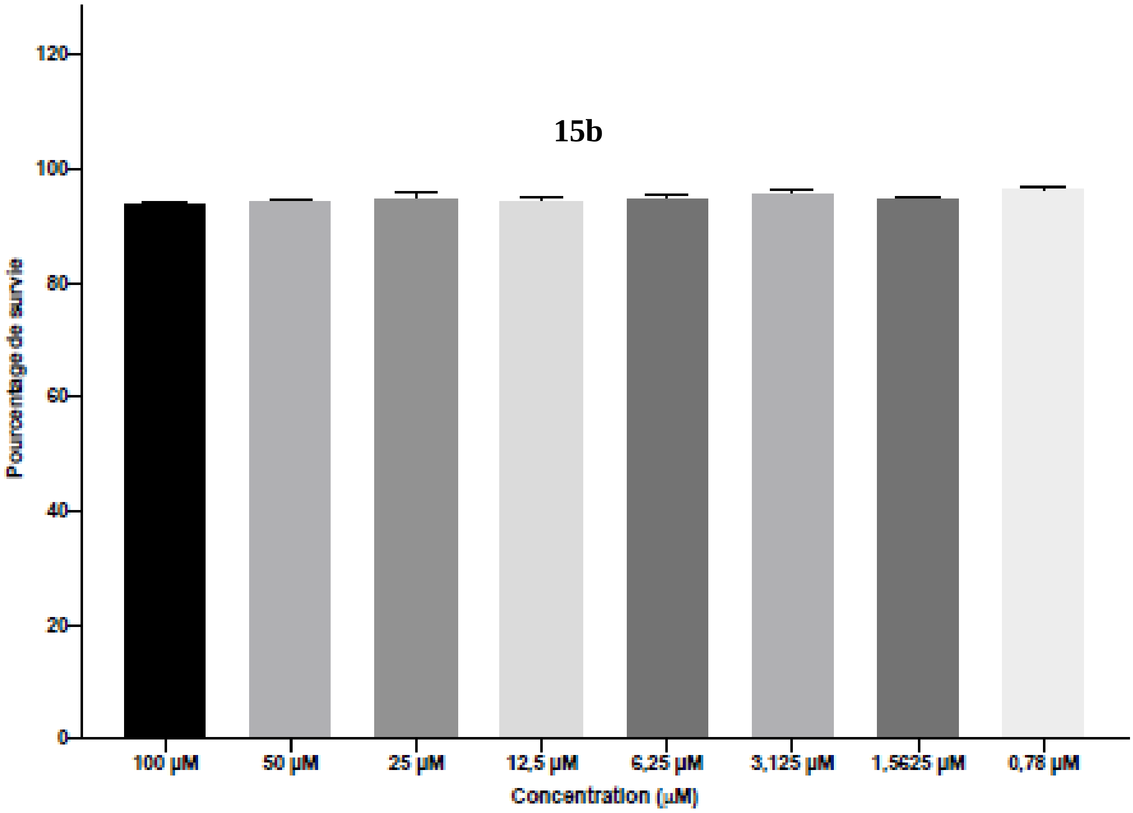
15b
Pourcentage de survie
0
20
40
60
80
100
120
100 µM
50 µM
25 µM
12,5 µM
6,25 µM
3,125 µM
1,5625 µM
0,78 µM
Concentration (µM)
